# FBXO7 sensitivity of phenotypic traits elucidated by a hypomorphic allele

**DOI:** 10.1371/journal.pone.0212481

**Published:** 2019-03-06

**Authors:** Carmen Ballesteros Reviriego, Simon Clare, Mark J. Arends, Emma L. Cambridge, Agnieszka Swiatkowska, Susana Caetano, Bushra Abu-Helil, Leanne Kane, Katherine Harcourt, David A. Goulding, Diane Gleeson, Edward Ryder, Brendan Doe, Jacqueline K. White, Louise van der Weyden, Gordon Dougan, David J. Adams, Anneliese O. Speak

**Affiliations:** 1 Wellcome Sanger Institute, Wellcome Genome Campus, Hinxton, Cambridgeshire, United Kingdom; 2 University of Edinburgh Division of Pathology, Centre for Comparative Pathology, Cancer Research UK Edinburgh Centre, Institute of Genetics & Molecular Medicine, Western General Hospital, Edinburgh, United Kingdom; University of Naples Federico II, ITALY

## Abstract

*FBXO7* encodes an F box containing protein that interacts with multiple partners to facilitate numerous cellular processes and has a canonical role as part of an SCF E3 ubiquitin ligase complex. Mutation of *FBXO7* is responsible for an early onset Parkinsonian pyramidal syndrome and genome-wide association studies have linked variants in *FBXO7* to erythroid traits. A putative orthologue in *Drosophila*, *nutcracker*, has been shown to regulate the proteasome, and deficiency of *nutcracker* results in male infertility. Therefore, we reasoned that modulating *Fbxo7* levels in a murine model could provide insights into the role of this protein in mammals. We used a targeted gene trap model which retained 4–16% residual gene expression and assessed the sensitivity of phenotypic traits to gene dosage. *Fbxo7* hypomorphs showed regenerative anaemia associated with a shorter erythrocyte half-life, and male mice were infertile. Alterations to T cell phenotypes were also observed, which intriguingly were both T cell intrinsic and extrinsic. Hypomorphic mice were also sensitive to infection with *Salmonella*, succumbing to a normally sublethal challenge. Despite these phenotypes, *Fbxo7* hypomorphs were produced at a normal Mendelian ratio with a normal lifespan and no evidence of neurological symptoms. These data suggest that erythrocyte survival, T cell development and spermatogenesis are particularly sensitive to *Fbxo7* gene dosage.

## Introduction

F box containing proteins form part of SCF E3 ubiquitin ligase complexes in addition to SKP1 and CULLIN. Within these complexes the F box subunits are essential for controlling the specificity of the targets proteins for ubiquitination [1]. FBXO7 is one such F box containing protein and within the SCF complex has been demonstrated to regulate ubiquitination of HURP [2], cIAP1 [3] and TRAF2 [4]. In addition to their role in the SCF complex, certain F box proteins can function via additional protein-protein interaction domains. Mutations in *FBXO7* have been linked with an early onset autosomal recessive Parkinsonian pyramidal syndrome [5]. In addition to loss-of-function mutations, two genome-wide associations studies have linked variants in *FBXO7* with several erythrocyte and stem cell traits [6, 7].

FBXO7 has been shown to interact with the proteasome inhibitor, PI31 [8]. Proteasomal regulation by the putative FBXO7 *Drosophila* orthologue, *nutcracker*, which was identified in a screen for genes affecting male fertility [9], was demonstrated to alter caspase activation and prevent spermatid individualisation [10]. Recently, FBXO7 has been demonstrated to alter proteasome activity leading to neuronal dysfunction [11], which could in part underlie the Parkinsonian pyramidal syndrome associated with loss-of-function mutations in *FBXO7*. However, an alternative mechanism has been proposed whereby FBXO7 can recruit PARKIN to the mitochondria and regulate mitophagy, such that mutated *FBXO7* results in increased dysfunctional mitochondria and neuronal cell dysregulation [12].

FBXO7 has also been implicated in cell cycle control and has been suggested to act as an oncogene [13]. FBXO7 can interact with CDK6 to regulate the activity of CYCLIN D/CDK6 complexes [13]. In addition, FBXO7 can also interact with the cell cycle inhibitor, CDKN1B (p27^Kip1^), and this regulation has been suggested to affect erythropoiesis in a murine model [14]. Furthermore, FBXO7 can regulate apoptosis through interaction with the apoptosis inhibitor cIAP1 and ubiquitination as part of the SCF complex [3].

Given the roles of FBXO7 within the SCF complex and its SCF-independent roles we sought to investigate the physiological roles of FBXO7. To do this we generated a *Fbxo7* hypomorphic allele, observing that male mice homozygous for this allele were infertile, similar to *Drosophila* carrying mutations in *nutcracker*, however these mice showed no evidence of shortened lifespan or any neurological symptoms. In addition, this hypomorphic allele resulted in regenerative anaemia due to a shortened erythrocyte half-life *in vivo* that was intrinsic to the haematopoietic lineage. These hypomorphic mice showed alterations to T cell phenotypes and were also highly susceptible to systemic infection with *Salmonella* Typhimurium, a phenotype that was neither T cell dependent nor regulated by other cells of the haematopoietic lineage. Thus, we confirm that erythrocytes and T lymphocytes are sensitive to *Fbxo7* gene dosage. We also reveal that hypomorphic male mice are infertile due to strongly impaired spermatogenesis, and highlight new roles for *Fbxo7* in regulating susceptibility to bacterial infection.

## Materials and method

### Mice

Generation of C57BL/6NTac *Fbxo7*^*tm1a(KOMP)Wtsi*^ (hereafter referred to as *Fbxo7*^*tm1a*^) mice was performed using ES cell clone EPD0622_3_D02 with genotyping performed as previously described [15] and are openly available (EMMA ID EM:06827). Mice carrying the *Fbxo7*^*tm1b*^ allele (whereby the critical exon was deleted by Cre recombinase) were generated by treating *Fbxo7*^*tm1a*^ two-cell embryos with cell permeable Cre as previously reported [16]. The *Fbxo7*^*tm1a*^ allele was converted to the floxed (conditional) *Fbxo7*^*tm1c*^ allele by crossing these mice with those ubiquitously expressing Flp recombinase from the *Rosa26* locus (FLPeR allele provided by Philippe Soriano [17] and targeting performed in JM8/F6 (C57BL/6N) embryonic stem cells to generate B6N-*Gt(ROSA)26Sor*^*tm1(FLP1)Dym*^*/J* mice). *Fbxo7*^*tm1c*^ mice were then bred to *Tg*^*CD4Cre*^ (B6.Cg*-Tg(Cd4-cre)1Cwi/BfluJ*, Jackson laboratory stock 022071) [18] in order to generate a T cell-specific deletion of *Fbxo7*. Animals were housed at a density of 2 to 6 mice per cage in polysulfone individually ventilated cages (Tecniplast) with sterilised Aspen bedding substrate and standard environmental enrichment, in a specific pathogen-free unit. The light cycle was maintained at 12h light/12h dark with lights off at 19:30 hours and no twilight period. Room temperature was 21 ± 2°C and humidity was regulated at 55 ± 10%. Mice received sterilized (vacuum packed and irradiated) diet (Lab Diets, 5021–3) from weaning and had *ad libitum* access to autoclaved water and food. In order to assess the fertility of homozygote (*Fbxo7*^*tm1a/tm1a*^) mice after confirmation of genotypes a single male was placed with a single fertile female for 11–16 weeks and signs of pregnancy assessed. All experiments were performed according to protocols approved by the UK Home Office regulations, UK Animals (Scientific Procedures) Act of 1986 and were approved by the Wellcome Sanger Institute Animal Welfare and Ethical Review Board.

### Gene expression and transcript sequence analysis

RNA was extracted from tissues using RNeasy fibrous or lipid kit (Qiagen Ltd, Manchester, UK) according to the manufacturer’s instructions. *Fbxo7* gene expression was assessed using FAM-conjugated TaqMan assays (Mm00462692_m1 for exons 3–4 spanning the inserted cassette for the tm1a allele or the downstream exon 5–6 assay Mm01240794_m1 for tm1b samples). Template RNA was added in duplex reactions in triplicate with endogenous control *B2m* VIC primer limited probe (Mm00437762_m1), using the EXPRESS One-Step Superscript qRT-PCR Kit (Thermo Scientific) and an Applied Biosystems Viia7 analyser. Relative gene expression between endogenous control and target genes were analysed using the ΔΔCT method [19]. For sequencing the targeted transcripts reverse transcriptase PCR was performed on RNA isolated from the liver of *Fbxo7*^*tm1a/tm1a*^ or *Fbxo7*^*tm1b/tm1b*^ mice using the Superscript IV one-step kit using the following primers Fbxo7_Exon3_F GGCCTAGTCAAAATGTTGAAGC and Fbxo7_Exon7_R TCCACAGCAGTGGGTCATT. The resulting product was sent for Sanger sequencing using the primers above.

### Western blotting

Brain, liver, spleen, kidney and testis were collected from a male WT and a *Fbxo7*^*tm1a/tm1a*^ male mouse. These samples were homogenised in 1 ml (spleen, kidney and testis) or 2 ml (brain and liver lobe) of radioimmunopreciptation assay buffer (10x stock from Merck) supplemented with 0.1% w/v sodium lauryl sulfate and protease inhibitors (cOmplete, Roche, Sigma-Aldrich, Poole, UK) in M tubes (Miltenyi Biotec, Bisley, UK) using a GentleMACS tissue dissociator and program protein_01. Lysates were placed on ice for 10 min and then insoluble material cleared by centrifugation at 15,000 x g for 15 min at 4°C. Protein levels in the cleared lysate were determined by a bicinchoninic acid protein assay (Pierce, ThermoFisher Scientific, Loughbourgh, UK) following the manufacturer’s protocol. A total of 50 μg protein was prepared for electrophoresis with NuPAGE LDS sample buffer and NuPAGE sample reducing agent (both Invitrogen, ThermoFisher Scientific) according to the manufacturer’s guidelines. Proteins were separated on a NuPAGE 4–12% Bis-Tris cell using MOPS running buffer together with Novex Sharp pre-stained protein ladder and MagicMark XP protein ladder and then transferred to PVDF membrane using the X-Cell II blot module and NuPAGE transfer buffer according to the recommended settings (all reagents from Invitrogen). After transfer the membrane was rinsed with tris buffered saline containing 0.05% Tween 20 (TBS-T) and blocked in 5% non-fat dried milk (Cell Signalling Technology, New England Biolabs (UK) Ltd, Hitchin, UK) prepared in TBS-T for 30 min at room temperature with gentle mixing. Primary rabbit polyclonal anti-FBXO7 antibody (Sigma-Aldrich SAB2100794) was diluted 1/500 in 2.5% non-fat dried milk in TBS-T and incubated overnight at 4°C with gentle mixing. The blot was washed with TBS-T prior to the addition of goat-anti rabbit IgG HRP conjugated antibody (Abcam, Cambridge, UK, ab97051, 1/5000 dilution in 2.5% non-fat dried milk in TBS-T) and incubated for 90 min at room temperature with gentle mixing. The blot was washed with TBS-T prior to application of chemiluminescent substrate (Western Bright ECL Spray, Advansta, Labtech, Uckfield, UK) and image acquisition with a LAS-4000 (GE Healthcare). The membrane was rinsed with TBS-T and incubated with stripping buffer (Restore PLUS stripping buffer, Pierce) then blocked for 30 min 5% non-fat dried milk prepared in TBS-T and cut between 60 and 80 kDa markers to be probed with two separate endogenous control antibodies (anti-β-actin, clone C4, Santa Cruz Biotechnology, Heidelberg, Germany, 1/500 or anti-vinculin, clone V284, Sigma-Aldrich, 1/10,000) for 90 min at room temperature with gentle mixing. After washing with TBS-T the blot was incubated for 60 min at room temperature with gentle mixing in secondary antibody (goat anti-mouse IgG HRP conjugated antibody, Abcam, ab97023, 1/5000 dilution in 2.5% non-fat dried milk in TBS-T). The blot was washed with TBS-T prior to image acquisition.

### Blood collection and analysis

Retro-orbital, tail vein or cardiac blood was collected into EDTA-coated tubes for haematology or heparinised tubes for plasma preparation (Kabe Labortechnik GmbH, Numbrecht, Germany). Complete blood counts were determined using a Vetabc system to provide red blood cell indices, white blood cell count and platelet count and volume parameters (Scil, Montpellier, France). Plasma was analysed for bilirubin, iron, aspartate aminotransferase, alanine aminotransferase, ferritin, amylase or lipase using an Olympus AU400 analyser (Beckman Coulter Ltd., High Wycombe, UK) with reagents supplied by Beckman Coulter or Randox (Randox Laboratories Ltd., Crumlin, UK).

### Bone marrow chimera generation

Wildtype (CD45.1 congenic B6.SJL^-^*Ptprc*^*a*^*Pepc*^*b*^*/*BoyJ, Jackson Laboratory strain ID 002014) mice were administered 2 x 5.4 Gy whole body irradiation from a gamma source 4 hours apart followed by tail vein administration of 4 x 10^6^ bone marrow cells from CD45.2 expressing wildtype or *Fbxo7*^*tm1a/tm1a*^ mice. Six weeks post-transplant a tail vein blood sample was taken from the mice to assess the relative proportions of CD45.1 (remaining host) versus CD45.2 (donor) cells by flow cytometry using PerCP-Cy5.5 conjugated anti-CD45.1 (clone A20, Biolegend, 0.4 μg/ml) and AlexaFluor 700 conjugated anti-CD45.2 (clone 104, BD Biosciences, 0.5 μg/ml). Mice were analysed 10 weeks after reconstitution.

### *Salmonella* Typhimruium challenge

Mice were infected intravenously with 0.2ml *Salmonella* Typhimurium M525 (phoN::tetC) containing 5x10^5^ CFU of bacteria in sterile phosphate buffered saline (Sigma-Aldrich). At various time points as indicated in the text, mice were sacrificed and the spleen and liver were harvested and blood collected via cardiac puncture under Isoflurane anaesthesia and placed into heparin tubes for the isolation of plasma. Prior to analysis (as above) plasma was filtered through 0.22 μm spin filters (Costar Spin-X) to eliminate any circulating bacteria. Organs were homogenised in sterile water and bacteria enumerated by serial dilution and plating onto agar plates (Oxoid). Mice were weighed daily throughout the infection and were humanely sacrificed if they lost more than 20% of their starting weight or were demonstrating clinical symptoms in accordance with UK Home Office regulations (United Kingdom Animals Scientific Procedures Act 1986).

### Histological analysis

Spleen, thymus and epididymis were fixed in neutral buffered formalin, embedded in paraffin and sections stained with haematoxylin and eosin according to standard methods. Spleen and liver from *Salmonella* infected mice were processed as above. Testes were collected and fixed in 4% paraformaldehyde (Sigma-Aldrich) prepared in 0.1M phosphate buffer pH 7.2 for 20 hours at 4°C. Testes were then postfixed with 1% osmium tetroxide, dehydrated and embedded in epoxy resin. Semithin sections (1 μm) were cut and stained with Toluidine Blue stain. To prepare epididymis smears the cauda epididymis were carefully collected and trimmed of adipose tissue. The epididymis was placed in a 35mm dish containing 0.5 ml of phosphate buffer saline and a number of small cuts made in the epididymal membrane and the sperm released with gentle swirling. The contents of the dish were visualised under a dissecting microscope and smears were prepared by applying 10 μl of this solution to one end of a microscope slide (Superfrost Plus, Thermo Scientifc) and using another slide at a 45^o^ angle to generate the film along the slide. After air drying the smears were stained with a modified Pappenheim stain (Hemacolor, Merck)

### Erythropoiesis analysis

Staining of single cell suspensions of spleen, bone marrow and whole blood with CD71, Ter119, CD45, Syto 16 and DAPI was performed as previously described [20]. In brief, single cells from spleen and bone marrow or blood (2 μl of whole blood added to 23 μl of normal 0.9% saline) were stained for 30 minutes at 4°C with the following antibodies: Ter119-APC (0.33 μg/ml, TER-119, Biolegend); CD71-PE-Cy7 (0.1 μg/ml, RI7217, Biolegend); and CD45-Alexa Fluor 700 (0.833 μg/ml, 30-F11, Biolegend) after blocking with 1 μg Mouse FC block (2.4G2, BD Biosciences) for 10 minutes. After washing the samples were incubated for 15 minutes at room temperature in 0.5 μM Syto 16 and 0.2 μg/ml DAPI (both Invitrogen) prepared in FACS buffer (Dulbecco’s phosphate buffered saline without calcium or magnesium (D-PBS, Gibco), supplemented with 1% bovine serum albumin (Sigma-Aldrich)). Samples were washed prior to acquisition on a BD LSRII instrument. Dead cells (DAPI^high^) and doublets (FSC-A vs FSC-H and SSC-H vs SSC-W) were excluded. Erythroid cells were identified as Ter119^+^ CD45^-^, with erythroblasts as CD71^+^ Syto 16^high^, reticulocytes as CD71^+^ Syto 16^low^ and mature erythrocytes as CD71^-^ Syto 16^neg^.

### *In vivo* clearance of erythrocytes

This was performed as described previously [21] with minor modifications. In brief, blood (700 μl) was collected under terminal anaesthesia from the retro-orbital sinus into EDTA coated tubes and washed twice with 14 ml of D-PBS. Blood from two mice of the same genotype were pooled and labelled with either 10 μM Vybrant CFDA (wild type) or 1 μM CellTracker Deep red (mutants, both Invitrogen) for 30 minutes at 37°C with constant gentle mixing by rotation. The reaction was quenched by the addition of 10 volumes of D-PBS containing 5% fetal bovine serum (FBS, Sigma). Erythrocytes were pelleted and washed twice with D-PBS prior to counting using a Vetabc system. Erythrocyte concentrations of each genotype sample were adjusted with D-PBS to 2 x 10^6^ erythrocytes/μl. The two genotypes were then pooled and 200 μl was injected into C57BL/6N recipient mice (10 weeks old; female) to transfuse 2 x 10^8^ erythrocytes/genotype. Blood samples (2 μl) were collected from the tail vein at the indicated time points and placed in 3 ml of flow cytometry buffer (D-PBS with 2mM EDTA and 0.5% FBS). The samples were mixed and acquired on a BD LSRII instrument with CFDA detected in the FITC channel (ex 488nm, em 530/30) and CellTracker Deep red in the APC channel (ex 633, em 660/20). Gates were set around the CFDA or CellTracker Deep red single positive erythrocytes and the percentage of total erythrocytes was determined. A total of 5,000 labelled erythrocytes were acquired and application settings in BD FACSDiva software were used to standardise the instrument voltage settings over the experiment duration. The percentage of the fluorescent erythrocytes was calculated as the percentage of the total labelled fraction determined from the blood collected 60 minutes after injection.

### T cell immunophenotyping

Blood was stained with titrated multicolour antibody panel for 20 minutes at room temperature prior to fixation with BD Cell Fix (BD Biosciences). Erythrocytes were lysed with BD PharmLyse (BD Biosciences) prior to washing with FACS buffer (D-PBS without calcium or magnesium, supplemented with 2mM EDTA, 0.5% fetal bovine serum and 0.1% sodium azide) and acquisition. Single cell suspensions of spleen or thymus were prepared by mechanical disruption using the frosted ends of microscope slides. Erythrocytes from spleen were removed via treatment with BD PharmLyse and passed through a 30-micron cell strainer (Miltenyi Biotec). Splenocytes or thymocytes were blocked with 1 μg Mouse FC block for 10 minutes at 4°C followed by the addition of titrated multicolour antibody panels and incubation at 4°C for 30 minutes. Samples were washed with D-PBS and viability staining with Fixable Viabilty Dye eFluor 455UV (Life Technologies) according to the manufacturer’s instructions. Samples were washed twice with FACS buffer prior to acquisition. Compensation was performed with antibody binding beads (UltraComp eBeads, eBioscience) and Amide Reactive beads (ArC beads, Life Technologies) using the automated compensation calculation in BD FACSDiva v8 using either a BD LSRII or BD LSRFortessa instrument. Samples were analysed in a blinded manner in FlowJo X (FlowJo, LLC). Details of antibodies used and concentrations are in S1 Table.

### Experimental design

All experiments performed on mice were not blinded to genotype due to this information being present on the cage cards. Experiments were performed in a randomised manner on a cage basis with controls and mutant mice generally being co-housed. Blood and tissue samples were processed in a blinded manner with the exception of the Salmonella counts. All analysis of flow cytometry data was performed in a blinded manner as was the histopathology. No *a priori* estimates were performed to calculate sample sizes for experiments and mice were allocated to treatment group by random allocation (Mendelian inheritance or via a predetermined allocation cage-based approach for bone marrow administration).

### Statistical analysis

All data was analysed in Prism v6 (Graph Pad) with an unpaired two-tailed students t test with Welch’s correction, unpaired two-tailed t test adjusted for multiple testing via the Holm-Sidak method for a family wise error of 5%, or two-way ANOVA with post-hoc test, as indicated in the figure legends.

## Results

### The *Fbxo7*^*tm1a*^ allele is hypomorphic

We generated mice that were homozygous for a ‘knockout first targeted’ EUCOMM/KOMP CSD *tm1a* allele of *Fbxo7* (*Fbxo7*^*tm1a/tm1a*^; S1 Fig; [22]) and found there was between 4 and 16% residual *Fbxo7* transcript present depending on the tissue tested (S1 Fig), indicating incomplete ablation of gene expression. The hypomorphic nature of the allele agrees with previous reports [14, 23]. Immunoblotting was performed using a commercially available antibody and, in some tissues, in particular testis, there was a reduction in the intensity of bands in the predicted size range for FBXO7 (S1 Fig). However, due to the numerous non-specific bands present with this primary antibody it is not possible to accurately conclude the degree of protein present in *Fbxo7*^*tm1a/tm1a*^ tissues. Homozygote *Fbxo7*^*tm1a/tm1a*^ mice were born at the expected Mendelian ratio (240 *Fbxo7*^*tm1a/tm1a*^ out of 1138 offspring from *Fbxo7*^*tm1/a+*^ intercrosses) and had a normal life span (S2 Fig). We then performed Cre conversion of the *Fbxo7*^*tm1a*^ allele to generate the *Fbxo7*^*tm1b*^ allele, in which the critical exon is excised, to produce a null allele [22]. Although *Fbxo7*^*tm1b/tm1b*^ pups were present at post-natal day 14 (6/43 homozygotes produced from *Fbxo7*^*tm1b/+*^ intercrosses) none of these survived weaning in agreement with a previous report using a similar allele [11].

Gene expression analysis of *Fbxo7*^*tm1b/tm1b*^ tissues using a qPCR assay spanning exons 5–6, downstream of the gene trap cassette, showed unexpected amplification of the target in liver and kidney samples (S3 Fig) despite the deletion of exon 4. Further analysis using RT-PCR and Sanger sequencing revealed that of the *Fbxo7* transcripts that were not truncated the remaining *Fbxo7*^*tm1b/tm1b*^ mRNA was interrupted by a 115-nucleotide sequence derived from the En2 exon in the splice acceptor region of the cassette (S3 Fig), a phenomenon which has been described previously using a similar IRES-βgeo gene trap cassette [24]. Although the removal of exon 4 is predicted to generate a frameshift on analysis the sequence of this product the inserted 115-nucleotides puts the resulting *Fbxo7* transcript back into frame. This has the potential to produce a protein lacking exon 4 and part of the annotated PI31 proteasome regulator domain but retaining the F-box domain and the regions which interact with PINK1 and CDK6 (S3 Fig), which could, in theory, have some dominant negative effect within cells. Similar analysis of RNA from *Fbxo7*^*tm1a/tm1a*^ indicated the presence of an in-frame full length transcript (S3 Fig) as suggested from the gene expression analysis.

### *Fbxo7*^*tm1a/tm1a*^ mice are anaemic

**Fig 1:**
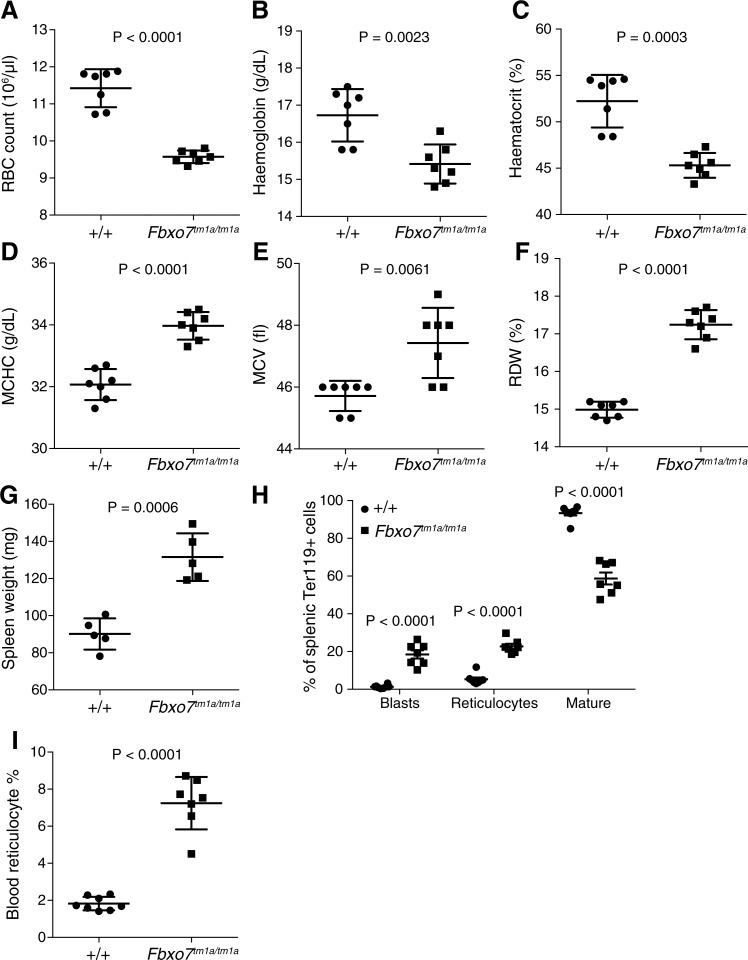
*Fbxo7* targeted mice present with anaemia and extramedullary haematopoiesis. (A) Red blood cell count (RBC); (B) haemoglobin; (C) haematocrit; (D) mean corpuscular haemoglobin concentration (MCHC); (E) mean corpuscular volume (MCV); (F) red blood cell distribution width (RDW); (G) spleen weight; (H) characterisation of splenic erythrocyte development; and (I) circulating reticulocyte; from 15–17 week old *Fbxo7*^*+/+*^ and *Fbxo7*^*tm1a/tm1a*^ female mice. P values calculated from unpaired two-tailed students t tests with Welch’s correction (A-G, I), or unpaired two-tailed students t tests with Holm-Sidak multiple testing correction (H). The data are representative of at least 3 independent experiments and each symbol represents an individual mouse with the line at the mean and error bars represent standard deviation or standard error of the mean for panel H.

Hypomorphic *Fbxo7*^*tm1a/tm1a*^ mice at 16 weeks of age showed a significantly reduced erythrocyte number, haemoglobin level and haematocrit with increased haemoglobin content and size of the erythrocytes, as well as an increased red blood cell distribution width (Fig 1A–1F). These altered erythrocyte indices indicate a macrocytic anaemia with anisocytosis that is hyperchromic, similar to that previously reported [14].

*Fbxo7*^*tm1a/tm1a*^ mice presented with splenomegaly suggestive of compensatory extramedullary haematopoiesis (Fig 1G). To quantify the extramedullary erythropoiesis in spleen we used flow cytometry and found a significant increase in the percentage of more immature (CD71+) cells. These immature cells were identified as both reticulocytes and erythroblasts (Fig 1H) and was accompanied by a significant increase in the circulating reticulocyte number (Fig 1I).

**Fig 2:**
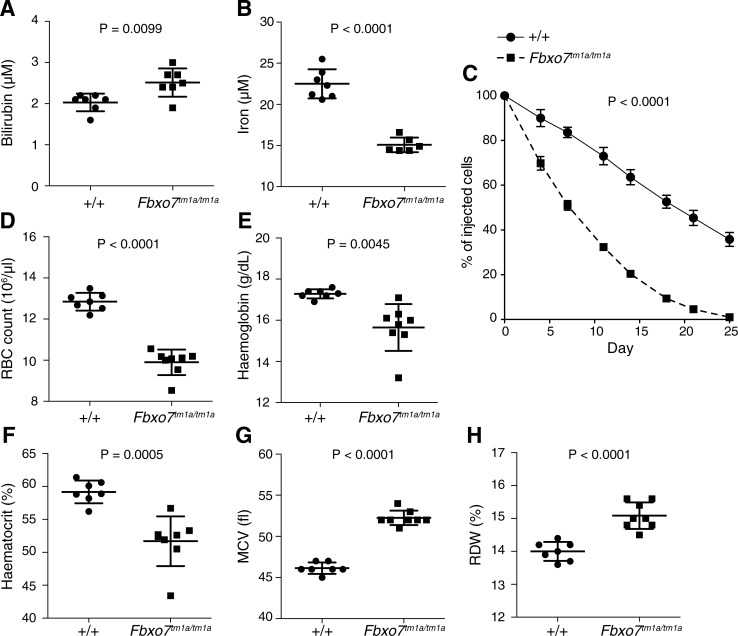
Anaemia in *Fbxo7* targeted mice is regenerative with a shorter erythrocyte half-life that is haematopoietic intrinsic. (A) plasma bilirubin and (B) plasma iron from 15-17-week-old *Fbxo7*^*+/+*^ and *Fbxo7*^*tm1a/tm1a*^ female mice. (C) *In vivo* half-life of *Fbxo7*^*+/+*^ and *Fbxo7*^*tm1a/tm1a*^ erythrocytes, the P value indicated is for each time point. D-H) characterisation of erythrocyte indices from *Fbxo7*^*+/+*^ and *Fbxo7*^*tm1a/tm1a*^ bone marrow chimeras 10 weeks after reconstitution (female recipient mice). All data representative of three independent experiments, each symbol represents an individual mouse with the line at the mean and error bars represent standard deviation, except for (C) where n = 6 for *Fbxo7*^*+/+*^ and *Fbxo7*^*tm1a/tm1a*^ with mean ± standard error of the mean. P values from an unpaired two-tailed students t test with Welch’s correction (A, B, D-H) or C two-way repeated measures ANOVA with Sidak post-hoc test for individual time points.

The anaemia and resulting increased erythropoiesis could be the result of a failure of erythrocyte development and/or due to shortened half-life of circulating erythrocytes. We assessed the circulating bilirubin concentration as a biomarker of erythrocyte destruction and observed that it was significantly increased in *Fbxo7*^*tm1a/tm1a*^ mice (Fig 2A) together with a decreased plasma iron level (Fig 2B) suggestive of a shortened erythrocyte half-life. In order to determine the actual erythrocyte half-life *in vivo* we adoptively transferred fluorescently labelled erythrocytes and observed a significantly reduced half-life of *Fbxo7*^*tm1a/tm1a*^ erythrocytes (Fig 2C). To eliminate possible environmental causes of the anaemia that were not intrinsic to the haematopoietic system we generated bone marrow chimeras and observed that these phenocopied *Fbxo7*^*tm1a/tm1a*^ mice (Fig 2D–2H).

### *Fbxo7*^*tm1a/tm1a*^ mice present with T cell abnormalities

**Fig 3:**
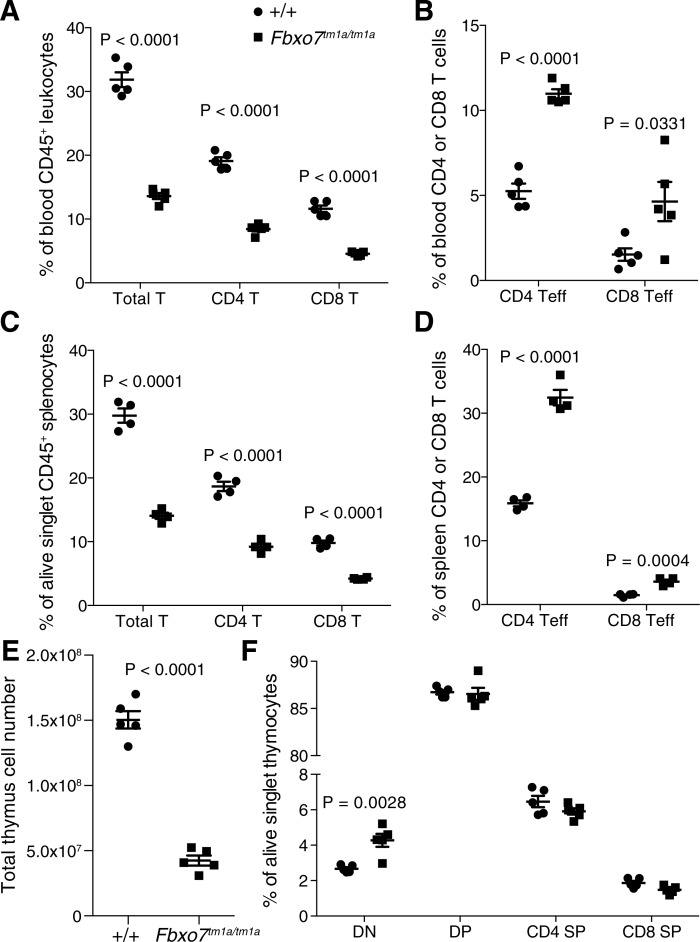
*Fbxo7*^*tm1a*^ mice have reduced circulating T cells with an altered phenotype and impaired T cell development. (A) Circulating T cell percentage in peripheral blood, (B) blood T cell effector percentage. (C) T cell percentage in the spleen and (D) splenic T cell effector percentage. (E) total thymus cell number and (F) T cell developmental stages in the thymus, DN = CD4/CD8 double negative, DP = CD4/CD8 double positive, CD4 SP = CD4 single positive and CD8 SP = CD8 single positive. All mice were female and 8 weeks old. P values from an unpaired two-tailed students t test with Holm-Sidak multiple testing correction except panel E which is from unpaired two-tailed students t tests with Welch’s correction. All data representative of three independent experiments, each symbol represents an individual mouse with the line at the mean and error bars represent standard error of the mean.

*Fbxo7*^*tm1a/tm1a*^ mice showed a significant decrease in circulating T cells in the blood, both in the CD4 and CD8 subsets (Fig 3A). The reduced number of T cells was accompanied by an altered phenotype of the remaining T cells with an increase in those expressing high levels of CD44 and low levels of L selectin (CD62L), representing an effector memory status (Fig 3B). The decreased number of T cells present in blood was also observed in the spleen (Fig 3C) with the increase in activated/memory T cells (Fig 3D). We next aimed to determine if there was a defect in T cell development as previously reported [25] and noticed severe thymic atrophy in *Fbxo7*^*tm1a/tm1a*^ mice, with the thymus virtually undetectable by 10 weeks of age. As would be expected the cellularity was greatly decreased (Fig 3E and S4 Fig) with an increase in most immature CD4/CD8 double negative T cells (Fig 3F), in agreement with the previous report.

**Fig 4:**
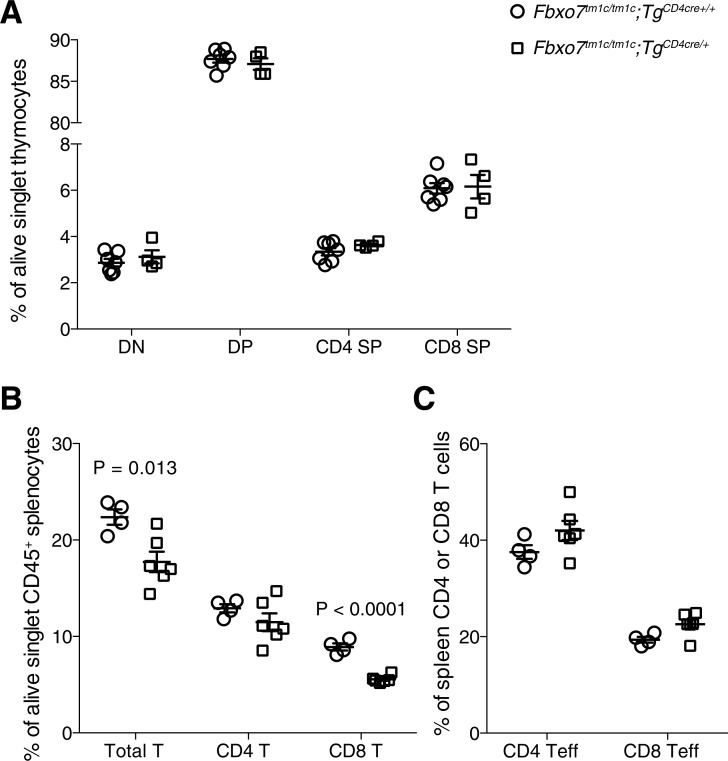
*Fbxo7* has a T cell intrinsic role in controlling T cell number in the periphery but not phenotype. (A) T cell developmental stages in the thymus, DN = CD4/CD8 double negative, DP = CD4/CD8 double positive, CD4 SP = CD4 single positive and CD8 SP = CD8 single positive. (B) T cell percentage in the spleen and (C) splenic T cell effector percentage. P values from an unpaired two-tailed students t test with Holm-Sidak multiple testing correction. All data representative of three independent experiments (spleen 16–20 week old female mice, thymus 12–14 week old male mice), each symbol represents an individual mouse with the line at the mean and error bars represent standard error of the mean.

We next sought to determine the role of FBXO7 in a T cell intrinsic manner via conversion of the *tm1a* allele into the floxed *tm1c* allele to allow for tissue specific ablation in T cells by crossing with mice carrying Cre under the control of the CD4 gene (*Fbxo7*^*tm1c/tm1c*^*;CD4-Cre* ^*+*^). Surprisingly in mice where *Fbxo7* was only deficient in the T cell lineage there was no evidence for thymic atrophy (S4 Fig) or any alterations in T cell development within the thymus (Fig 4A). This could be associated with the fact that under the control of a CD4 transgene, *Cre* is known to be expressed in the late double-negative stage [26] and thus there is likely to be insufficient time for any thymic phenotype to be observed. In the spleen, there was a mild reduction in T cell number in the mice where *Fbxo7* was specifically deleted within T cells that was mainly driven by the CD8 compartment which experienced a similar decrease as that of the whole body *Fbxo7* deficient (*Fbxo7*^*tm1a/tm1a*^) mice (Fig 4B). However, in contrast to the whole-body deficient animals there was no alteration in the phenotype of the T cells in the specific T cell deletion (Fig 4C). This would suggest that the thymic atrophy and T cell phenotypes are not regulated in a T cell intrinsic manner.

### *Fbxo7*^*tm1a/tm1a*^ mice are susceptible to *Salmonella* infection

**Fig 5:**
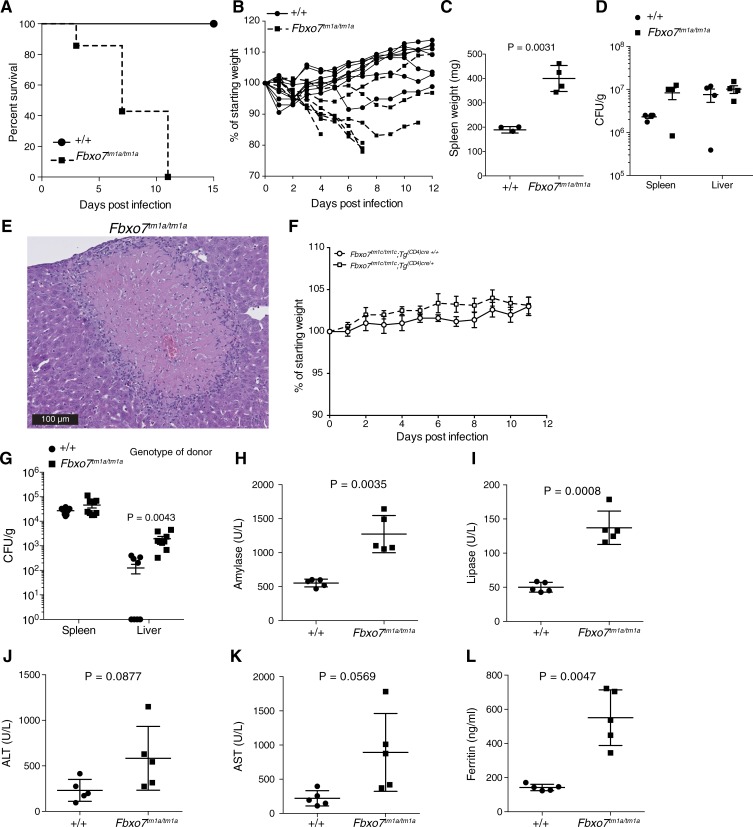
Increased morbidity after infection of *Fbxo7*^*tm1a*^ mice with *Salmonella* typhimurium. (A) Survival curve of 4 female and 4 male *Fbxo7*^*+/+*^ mice and 3 female and 4 male *Fbxo7*^*tm1a/tm1a*^ mice. (B) body weight changes during the course of infection from mice shown in panel A. (C) spleen weights at day 6 post Salmonella infection of male mice, with 3 *Fbxo7*^*+/+*^ and 4 *Fbxo7*^*tm1a/tm1a*^ mice. (D) spleen and liver *Salmonella* counts at day 6 post infection of male mice, with 3 *Fbxo7*^*+/+*^ and 4 *Fbxo7*^*tm1a/tm1a*^ mice. (E) histopathological image of liver showing a typical necro-inflammatory focus with an oval nodule consisting of eosinophilic necrotic hepatocytes surrounded by a rim of inflammatory cells that are predominantly neutrophil leucocytes, from a male *Fbxo7*^*tm1a/tm1a*^ mouse at day 6. (F) body weight changes during course of infection for male mice deficient for *Fbxo7* in T cells (*Fbxo7*^*tm1c/tm1c*^*;CD4-Cre* ^*+*^ 8 mice) and littermate controls (*Fbxo7*^*tm1c/tm1c*^*;CD4-Cre—*5 mice). (G) spleen and liver *Salmonella* counts at day 14 post infection from irradiated female mice reconstituted with *Fbxo7*^*+/+*^ (10 mice) or *Fbxo7*^*tm1a/tm1a*^ bone marrow (9 mice). (H-L) plasma clinical chemistry parameters at day 6 post infection from male mice (5 mice/genotype) for plasma levels of amylase, lipase, alanine aminotransferase (ALT), aspartate aminotransferase (AST), and ferritin (P values calculated from unpaired two-tailed students t tests with Welch’s correction). For panels C, D and G-L symbols represent individual mice with the line at the mean and error bars representing the standard error of the mean (D and G) or standard deviation (C, H-L). For panel F symbols represent the mean with error bars representing the standard error of the mean.

As part of a phenotype-screening programme, mice were given a systemic infection with *Salmonella* Typhimurium that is normally sublethal in wild-type C57BL6/N mice. In contrast, the *Fbxo7*^*tm1a/tm1a*^ mice needed to be humanely sacrificed at an early time point (prior to day 11) due to exacerbated weight loss (reached 20% limit) or displaying health concerns (Fig 5A and 5B). We repeated the assay collecting samples at various times post-infection and assessed bacterial burden. The splenomegaly present in uninfected mice was exacerbated by the infection (Fig 5C). There was a slight increase in the bacterial burden in the spleen but this was not significant with no difference in the liver at day 6 post-infection (Fig 5D). The livers of infected *Fbxo7*^*tm1a/tm1a*^ mice presented with macroscopic alterations and histologically showed necro-inflammatory foci with oval nodules of hepatocyte necrosis surrounded by inflammatory cell infiltration (Fig 5E) that were not seen in infected control livers. T cells have been demonstrated to have an important role in mediating the complete clearance of *Salmonella* infection [27] and given the T cell abnormalities in these mice, we sought to determine the role of FBXO7 within T cells. Using T cell-specific *Fbxo7* deficient mice (*Fbxo7*^*tm1c/tm1c*^*; CD4-Cre* ^*+*^) we repeated the *Salmonella* infection but observed no weight loss phenotype indicating that there is no role of FBXO7 within mature peripheral T cells for the phenotype observed (Fig 5F). We next generated bone marrow chimeras to elucidate if the susceptibility to *Salmonella* was driven by the haematopoietic system. The bone marrow chimeras had no overt phenotype following *Salmonella* infection, except possibly a mild impairment in *Salmonella* clearance from the liver at day 14 post infection (Fig 5G), suggesting the susceptibility is driven by a non-haematopoietic lineage. With the histological changes, we investigated if the morbidity was linked to a toxic response to *Salmonella* infection similar to that observed in mice deficient in vitamin B12 through targeted mutation in gastric intrinsic factor [28]. Assessing plasma concentrations of various constituents in homozygous *Fbxo7*^*tm1a*^ mice, we observed an increase in circulating amylase and lipase (Fig 5H and 5I) suggestive of pancreatic damage. This was accompanied by a trend to increased levels of hepatic enzymes, alanine aminotransferase (ALT, P = 0.0877, Fig 5J) and aspartate aminotransferase (AST, P = 0.0569, Fig 5K), which are typically released after liver damage. There was also a striking increase in ferritin, an acute phase protein (Fig 5L). Together these data suggest that FBXO7 is required for the normal response to systemic *Salmonella* infection.

### *Fbxo7*^*tm1a/tm1a*^ male mice are infertile

**Fig 6:**
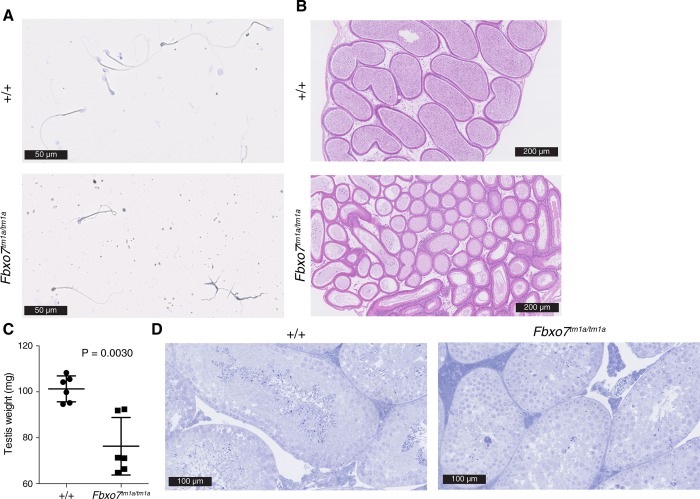
Defects in the testis and epididymis of *Fbxo7*^*tm1a/tm1a*^ mice. (A) Image of epididymal spermatozoa smear stained with hemacolor from 12-14-week-old mice. (B) H&E stained section of epididymis from 20-week-old mice. (C) testis weight from 18-19-week-old mice. (D) semithin toloudine blue stained section image of testis from 8-week-old mice. Images are representative of at least two mice per genotype, P value from an unpaired two-tailed students t test with Welch’s correction with each symbol representing a testis, line is at the mean and error bars represent standard deviation.

It has been suggested that *Fbxo7* is the mammalian orthologue of the *Drosophila*, *nutcracker*. Flies with a mutation in *nutcracker* are sterile due to a defect in spermatid individualisation [9] associated with reduced proteasome activity [10]. Perhaps surprisingly given the hypomorphic nature of the *Fbxo7*^*tm1a*^ allele we observed a similar sterility in homozygous male mice with no litters resulting from four *Fbxo7*^*tm1a/tm1a*^ male mice paired with known fertile females. We sought to determine if mature spermatozoa were released from the testis into the epididymis and thus harvested the epididymis and isolated the contents. Visualising the contents of the epididymis under a microscope showed a greatly reduced spermatozoa count from *Fbxo7*^*tm1a/tm1a*^ mice and the few spermatozoa that were present had an abnormal morphology (Fig 6A) with no motility. On histological examination of the epididymis they were devoid of spermatozoa in *Fbxo7*^*tm1a/tm1a*^ mice compared to WT mice (Fig 6B) In agreement with a developmental defect the weight of the testis was reduced in *Fbxo7*^*tm1a/tm1a*^ mice (Fig 6C) and there was aberrant spermatogenesis with spermatid arrest and markedly reduced spermatozoa formation observed in sections of the testis (Fig 6D).

## Discussion

In studying a hypomorphic allele we have been able to investigate the pleiotropic functions of FBXO7 in a murine model. With 4–16% residual gene expression remaining there is no evidence of Parkinson’s-like symptoms or a shortened lifespan. However, a shortened erythrocyte half-life with concomitant regenerative anaemia is observed, together with male infertility due to a lack of normal mature spermatozoa. In addition, a T cell developmental defect is present and the mice are susceptible to morbidity after systemic infection with *Salmonella* Typhimurium.

FBXO7 has been demonstrated to interact with numerous other proteins including SKP1 and CULLIN to form an E3 ubiquitin ligase complex, with Parkin and PINK1 to regulate mitophagy, with CDK6 and p27 to influence cell cycle progression and PI31 to regulate the proteasome. It is believed that these different interactions can underlie the pleiotropic phenotypes observed. The interaction with Parkin and PINK1 has been suggested to be critical to the observed Parkinsonian pyramidal syndrome with a defect in mitophagy resulting in dopaminergic neuronal cell death [12]. However, recent studies also suggest that proteasomal regulation by FBXO7 also contributes to neuronal cell survival [11]. This proteasomal regulation has also been linked with the male infertility observed in *nutcracker* deficient flies, with *nutcracker* suggested to be the *Drosophila Fbxo7* orthologue. This reduced proteasome activity leads to a build-up of dBruce, an inhibitor of caspase activity, that prevents the non-apoptotic caspase activity required for the individualisation of mature spermatids within the testis and their release as spermatozoa into the epididymis [10]. Similar to *nutcracker* flies we observed a similar defect in the hypomorphic *Fbxo7*^*tm1a/tm1a*^ male mice, which were infertile due to a lack of normal mature spermatozoa in the epididymis.

In contrast, the shorter erythrocyte *in vivo* half-life is more likely to be attributed to the impaired mitochondrial activity observed in *Fbxo7* deficient erythroid lineage cells leading to increased cytosolic reactive oxygen species (ROS) [14]. Other mutations that result in increased ROS, such as those affecting AMPK, have been demonstrated to give rise to shortened erythrocyte half-life and regenerative anaemia [29]. Given the shorter half-life it is hard to disentangle if there is also a developmental defect within the erythroid lineage, which could be possible given evidence that caspases play important roles in erythroid enucleation and terminal differentiation [30]. Further, it has previously been demonstrated that FBXO7, via interaction with p27, can modulate erythroblast differentiation [14]. Genome wide association studies have linked *FBXO7* variants to mean corpuscular volume and mean corpuscular haemoglobin traits [6, 7] and this study adds further evidence to the potential mechanism by which FBXO7 could regulate erythrocyte development, differentiation and survival.

Using a combination of ubiquitous targeting of *Fbxo7* (*Fbxo7*^*tm1a/tm1a*^) and T cell-specific deletion of *Fbxo7* (*Tg*^*CD4cre*^) we have investigated the role for FBXO7 within the T cell lineage. When *Fbxo7* is deficient ubiquitously the number of T cells is decreased and there is an increase in T cells showing an activated phenotype. These mice also have severe thymic atrophy with the thymus undetectable after approximately 10 weeks of age. In contrast, while there is a T cell intrinsic role for *Fbxo7* with regard to determining the number of T cells, perhaps through regulation of the cell cycle and proliferation, it however does not influence the phenotype of the cells. In contrast, the increased activated T cells could be linked to the shorter erythrocyte half-life and regenerative anaemia as it is only present in the whole body *Fbxo7*^*tm1a*^ mice which would suggest it is triggered by the environment rather than an intrinsic role for FBXO7 within T cells.

The phenotype after systemic infection with *Salmonella* is not likely to be attributed to a defect in the ability to control the bacteria as although the burden within the spleen and liver was increased it was not to the same degree as other murine models with an innate defect in the control of intracellular pathogens [27]. This is supported by the fact that neither *Fbxo7* T cell deficient mice nor bone marrow chimeras show a similar phenotype, suggesting that this effect is not mediated by a haematopoietic cell lineage. Instead, this is likely associated with a toxic syndrome given the pathological and physiological alterations we observed, such as the increased markers of liver and pancreatic damage with elevated levels of ALT, AST, amylase and lipase. This could potentially be mediated by FBXO7 directed ubiquitination of cIAP1 and TRAF2 and regulation of NF-κB signalling [4] or another via interaction between FBXO7 and other binding partners.

In conclusion, this study has utilised a hypomorphic allele to elucidate the differential contribution of FBXO7 in various cellular lineages. Given the phenotype observed in erythrocyte survival and spermatogenesis, it would appear that these cell types are more sensitive to a reduction in cellular *Fbxo7* expression levels where even 4–16% is not sufficient to maintain normal function. In contrast, the lack of an overt neurological defect, in agreement with another study [11], suggests that a complete ablation of *Fbxo7* is required for the development of this phenotype and that in this case, if not restricted to the neurons, results in post-natal lethality.

## Supporting information

S1 Table Details of fluorochrome conjugated antibodies used in the study for immunophenotyping spleen, blood and thymus. (XLSX)

S1 Fig Molecular characterisation of *Fbxo7*^*tm1a*^ allele. (A) Diagrammatic representation of the *Fbxo7*^*tm1a*^ allele. (B) RNA expression of *Fbxo7* in *Fbxo7*^*+/+*^ and *Fbxo7*^*tm1a/tm1a*^ brain, liver, spleen, kidney and testis samples. Data is presented as the relative expression of three samples per genotype from male mice aged 18 weeks old, showing the mean and 95% confidence intervals using a probe spanning exons 3–4. (C) Protein expression of FBXO7 and two endogenous controls (VINCULIN and β-ACTIN) in brain, liver, spleen, kidney and testis tissue lysates from 20-week-old *Fbxo7*^*+/+*^ and *Fbxo7*^*tm1a/tm1a*^ male mice. The dotted box indicates the suggested size of FBXO7 isoforms and * indicates endogenous immunoglobulin heavy and light chain detected by the anti-mouse IgG-HRP conjugate.
(EPS)

S2 Fig Survival of *Fbxo7*^*+/+*^ and *Fbxo7*^*tm1a/tm1a*^ mice. *Fbxo7*^*+/+*^ (8 female and 15 male) and *Fbxo7*^*tm1a/tm1a*^ mice (7 female and 14 male), P = 0.9409 Mantel-Cox test.
(EPS)

S3 Fig Gene expression in *Fbxo7*^*tm1b/tm1b*^ mice. (A) RNA was isolated and *Fbxo7* expression determined in kidney and liver from *Fbxo7*^*tm1b/tm1b*^ mice and wild-type controls (three mice per genotype) showing the mean and 95% confidence intervals using a probe spanning exons 5–6. (B) Schematic of *Fbxo7*^*tm1b*^ allele. (C) RT-PCR and Sanger sequencing across the targeted region. (D) Schematic of *Fbxo7* exons and key protein domains from wild-type full length and from the two transcripts generated in *Fbxo7*^*tm1b/tm1b*^ mice. (E) Schematic of *Fbxo7*^*tm1a*^ allele. (F) RT-PCR and Sanger sequencing across the targeted region.
(EPS)

S4 Fig Histology of thymus. (A) *Fbxo7*^*+/+*^ and *Fbxo7*^*tm1a/tm1a*^ mice (25 weeks old). (B) *Fbxo7*^*tm1c/tm1c*^*;CD4-Cre—*(control) *Fbxo7*^*tm1c/tm1c*^*;CD4-Cre* ^*+*^ (T cell specific *Fbxo7* deletion) mice (20 weeks old). Images are representative of at least two mice per genotype.
(TIF)
