# Supplementary figures and images for: FBXO7 sensitivity of phenotypic traits elucidated by a hypomorphic allele
Source: PLoS One. 2019 Mar 6;14(3):e0212481. doi: 10.1371/journal.pone.0212481 (PMC6402633; doi:10.1371/journal.pone.0212481)

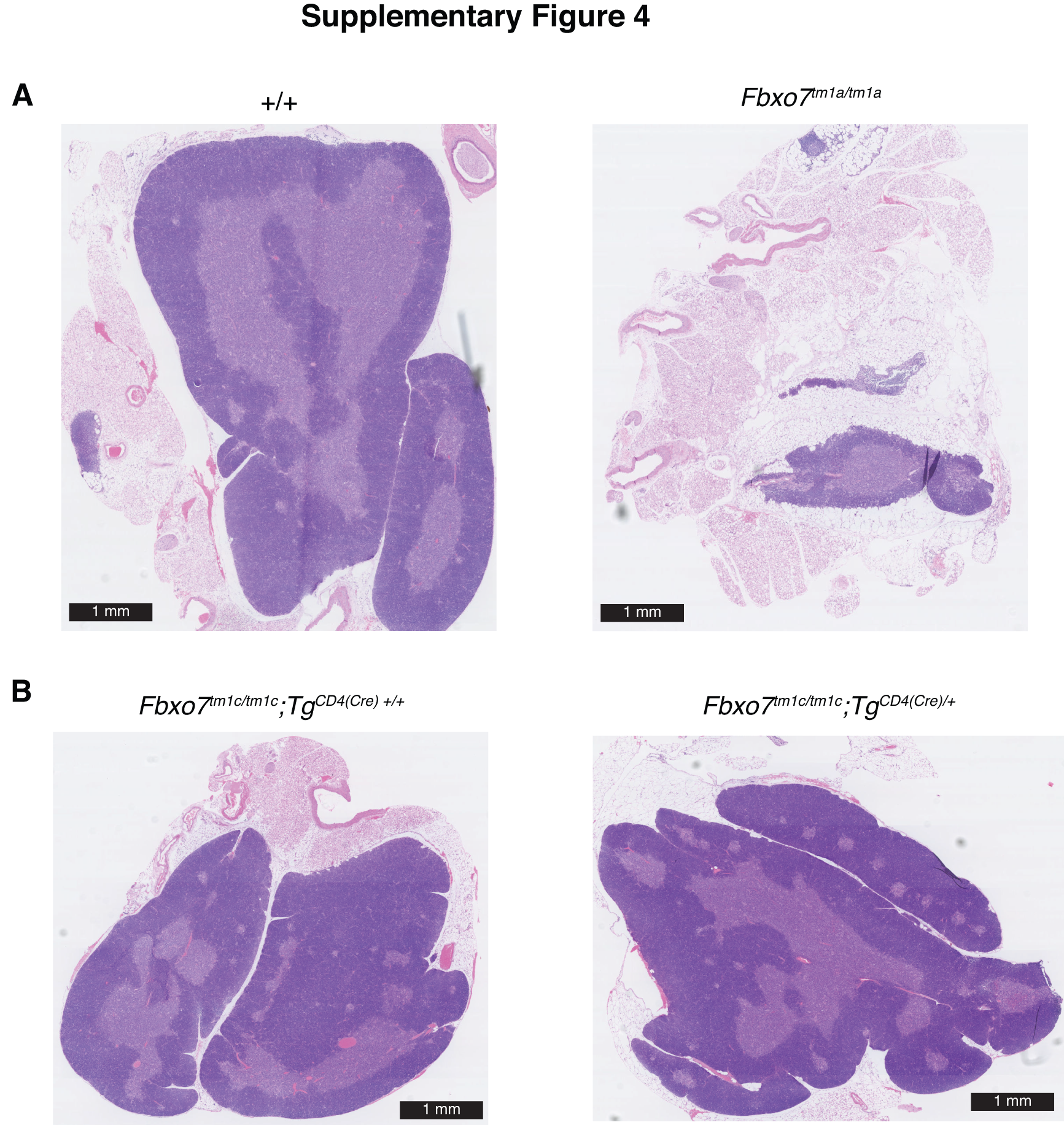

Supplement: S4 Fig — (A) Fbxo7+/+ and Fbxo7tm1a/tm1a mice (25 weeks old). (B) Fbxo7tm1c/tm1c;CD4-Cre—(control) Fbxo7tm1c/tm1c;CD4-Cre + (T cell specific Fbxo7 deletion) mice (20 weeks old). Images are representative of at least two mice per genotype. (TIF) [file pone.0212481.s005.tif]
